# Looking beyond silybin: the importance of other silymarin flavonolignans

**DOI:** 10.3389/fphar.2025.1637393

**Published:** 2025-07-21

**Authors:** Michal Selc, Andrea Babelova

**Affiliations:** ^1^ Centre for Advanced Material Application, Slovak Academy of Sciences, Bratislava, Slovakia; ^2^ Department of Nanobiology, Cancer Research Institute, Biomedical Research Center, Slovak Academy of Sciences, Bratislava, Slovakia

**Keywords:** silymarin, silybin, flavonolignans, natural products, research bias

## Abstract

Silymarin, an extract from the seeds of milk thistle (*Silybum marianum*), has been widely studied and used for its hepatoprotective and pharmacological properties. For decades, both experimental and clinical research has been predominantly focused on a single component, silybin, while other related flavonolignans, such as silychristin, isosilybins, silydianin, dehydrosilybin, and the flavonoid taxifolin, have been understudied. However, these less known components may possess unique or even superior pharmacological activities compared to silybin, including strong antioxidant, anti-inflammatory, antiviral, and selective anticancer effects. Exploring these other constituents beyond silybin may unlock new opportunities for drug discovery and personalized phytotherapy, ultimately advancing the development of next-generation flavonolignan-based therapeutics.

## 1 Introduction: silybin, a major flavonolignan from silymarin

Silymarin, an extract from the seeds of *Silybum marianum* (milk thistle), has been used for its hepatoprotective, antioxidant, anti-inflammatory, and anticancer properties for over 2000 years. Its therapeutic potential has traditionally been attributed to silybin (also known as silibinin), the major and most extensively studied component of the silymarin complex in pharmacological research. Silybin is an equimolar mixture of silybin A and silybin B, which differ in stereochemistry and may exhibit distinct biological activities when studied individually. Isolated and structurally characterized in the 1960s and 1970s, silybin quickly became the primary focus of pharmacological research on milk thistle (Wu et al., 2024).

The importance of silybin has been reflected in therapeutic applications. Legalon SIL, a water-soluble intravenously administered formulation of a silybin derivative, is approved in several countries to treat *Amanita phalloides* intoxication (Mengs et al., 2012). More recently, silybin-based products are currently being investigated for a broader spectrum of liver-related and metabolic disorders. One example is Realsil, a complex of silybin and phosphatidylcholine developed to enhance the oral bioavailability of silybin, which is poorly soluble. These complexes aim to overcome silybin’s pharmacokinetic limitations and are currently undergoing phase III clinical trials for indications such as metabolic dysfunction-associated steatohepatitis (Loguercio et al., 2012).

This intense scientific and clinical focus on silybin has, however, resulted in a research bottleneck. Although silybin is undoubtedly an important compound, it has become the dominant representative of silymarin in the literature. Its pharmacokinetics, molecular targets, and mechanisms of action rank among the most extensively studied of any plant-derived flavonolignan. Consequently, other structurally related flavonolignans and flavonoid taxifolin, some of which demonstrated equal or even superior bioactivity in specific pharmacological models, including anti-inflammatory, antioxidant, and cytotoxicity assays, have remained largely overlooked. In this perspective, we revisit the central role of silybin in silymarin research and question whether this narrow focus has limited the exploration of other potentially valuable constituents. With growing evidence that compounds such as 2,3-dehydrosilybin, silychristin, or isosilybin B may possess distinct and therapeutically relevant properties, it may be time to shift the research paradigm and broaden our scientific lens.

## 2 The other flavonolignans: pharmacological potential beyond silybin

**FIGURE 1:**
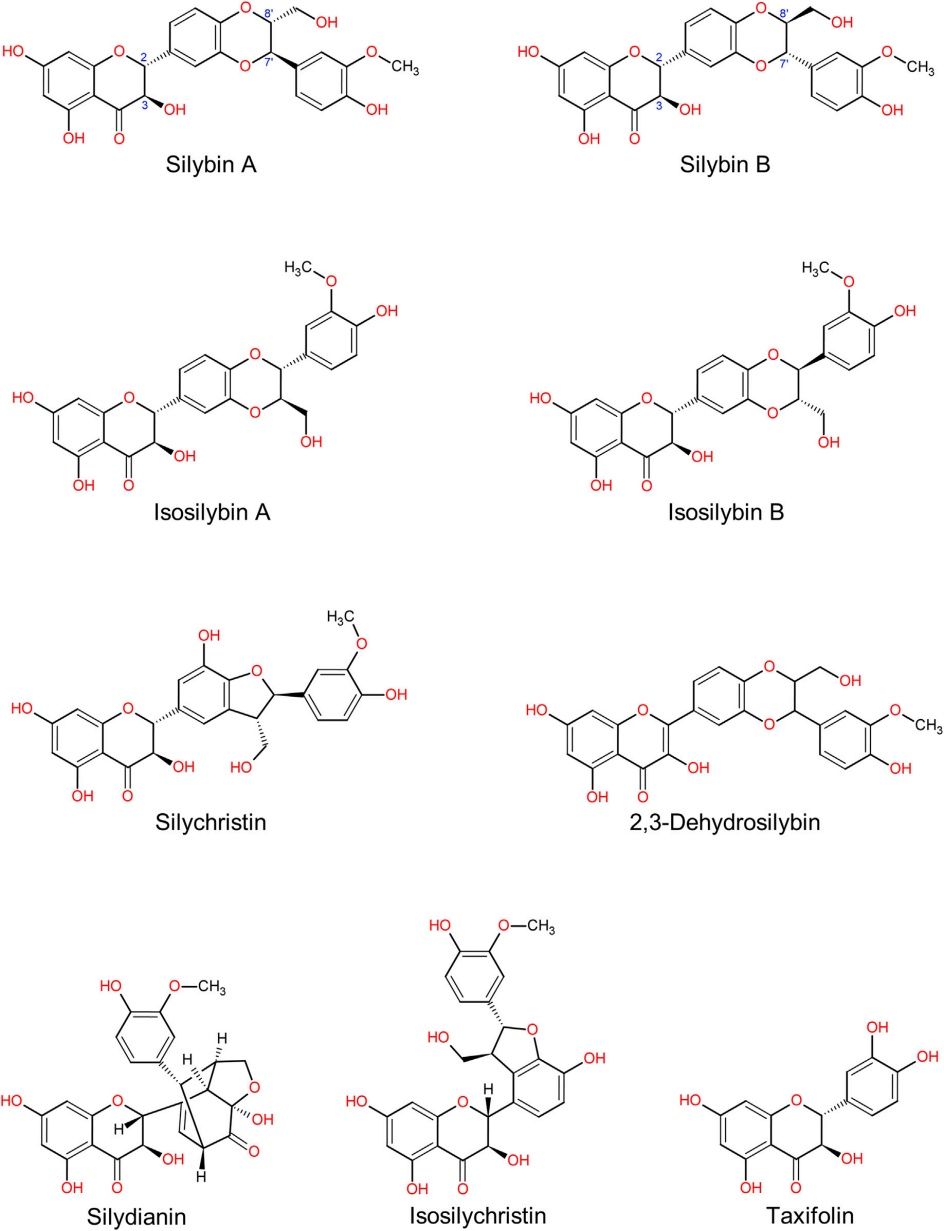
The main compounds of silymarin, an extract from the seeds of Milk thistle (Silybum marianum). Silybin A and silybin B together constitute silybin, the principal component of silymarin (40%–60%). Molecular structures were drawn using ACD/ChemSketch software based on data obtained from the PubChem database.

While silybin has received most of the attention in silymarin research, it is only one of several flavonolignans present in the extract. Silybin constitutes approximately 40%–60% of the total flavonolignan content in standardized silymarin preparations (Fenclova et al., 2019). This natural abundance has significantly contributed to its dominant role in both basic and clinical research. Other major constituents include silychristin (15%–25%), isosilybin A (10%), silydianin (5%–10%), isosilybin B (5%), 2,3-dehydrosilybin (<5%), taxifolin (<5%) and isosilychristin (<3%) (Figure 1) (Fenclova et al., 2019). These values are based on HPLC analyses of standardized seed extracts, but some variability may still occur depending on the extraction method and analytical approach used (Kim et al., 2024).

Several silymarin flavonolignans, such as silybin A (as a diastereomer), silybin B (as a diastereomer), isosilybin A or isosilybin B share a common flavonolignan skeleton (C_25_H_22_O_10_) but differ in stereochemistry and functional groups. Diastereoisomeric flavonolignans differ in the absolute configuration at the C-7′ and C-8′ positions, as demonstrated by X-ray crystallography and NMR spectroscopy (Lee and Liu, 2003). These stereochemical variations may underlie the distinct biological activities. In addition to stereochemistry, other structural features modulate the biological activity of individual flavonolignans. For instance, the presence of ortho-dihydroxy groups on the B-ring, as seen in taxifolin, contributes to antioxidant capacity, or a double bond between C-2 and C-3, as seen in 2,3-dehydrosilybin, enhances radical scavenging and strengthens antioxidant activity (Rice-Evans et al., 1996; Pyszková et al., 2016). These oxidation-related modifications increase the planarity of the molecule, potentially affecting its interactions with cellular targets. Such structure–activity relationships (SAR) are crucial for understanding the diverse pharmacological effects of silymarin constituents and for guiding their potential therapeutic applications.

These SAR are particularly prominent in certain lesser-studied silymarin constituents. For example, 2,3-dehydrosilybin and silychristin exhibit stronger cytoprotective and antioxidant activity than silybin at equivalent concentration (Jurčacková et al., 2025). Similarly, isosilybin A and isosilybin B were reported nearly 2 decades ago to selectively induce apoptosis and cause pronounced G1 arrest in prostate cancer cells, while sparing nonmalignant counterparts (Deep et al., 2007). This degree of tumor-specific targeting has not been demonstrated for silybin to date. In fact, one study even reported the opposite effect, with silybin exhibiting greater toxicity toward non-tumor hepatocytes than toward hepatoma cells (Şumnulu, 2023). In a comprehensive study by Polyak et al. (2010), all major silymarin flavonolignans and the flavonoid taxifolin were tested side by side across multiple hepatoprotective assays, including antiviral activity, NF-κB inhibition, antioxidant function, and T-cell immunomodulation. Compounds such as isosilybin A and silybin A (as an isolated diastereomer) outperformed silybin in several assays, particularly in antiviral, anti-inflammatory and T-cell proliferation activity. Taxifolin also demonstrated strong antiviral and antioxidant effects, although it lacked immunomodulatory activity in the T-cell assay. Notably, isosilybin B exhibited cytotoxicity in human hepatoma cells at concentrations above 10 μM, whereas all other tested compounds were well tolerated up to 80 µM. These findings underscore the potential of underrepresented constituents and challenge the prevailing assumption that silybin is the sole bioactive driver of silymarin’s effects (Polyak et al., 2010). A detailed comparison of antioxidant properties among the main silymarin components further revealed that taxifolin, silychristin, and silydianin all exhibited stronger radical scavenging activity than silybin in DPPH, ORAC, and ABTS^+^ assays, with taxifolin showing by far the most potent effect - its EC_50_ values were approximately 10-fold lower than those of silybin (Anthony and Saleh, 2013; Kim et al., 2024). Despite representing only <5% of the total silymarin mixture, taxifolin may contribute disproportionately to the extract’s overall antioxidant effects.

## 3 Why the scientific focus remained on silybin

The disproportionate research focus on silybin can be attributed to a combination of historical, technical, and commercial factors. Historically, the focus on silybin was support by its relative abundance in silymarin and by the fact that it was technically the easiest to isolate in pure form. In contrast, other flavonolignans occurred in lower concentrations and were difficult to purify, often requiring complex and low-yield procedures that hindered broader investigation (Křenek et al., 2014; Valentová et al., 2018). However, recent analytical advances have enabled more precise separation of individual flavonolignans, including their closely related stereoisomers. Notably, a capillary electrophoresis method developed by Riasová et al. (2022) achieved baseline resolution of all major flavonolignans in silymarin, including the diastereomeric pairs silybin A/B and isosilybin A/B, demonstrating that full separation is feasible under optimized conditions (Riasová et al., 2022).

As the first flavonolignan to be isolated, structurally characterized, and extensively studied, silybin naturally became the scientific prototype for silymarin-related research (Pelter and Hänsel, 1968). Notably, it took nearly a decade after the structural elucidation of silybin for the first publication focusing on other flavonolignans - such as silychristin or silydianin - to appear (Greimel and Koch, 1977). In that interval, at least 15 additional studies on silybin were published, further reinforcing its position as the central focus of silymarin research. By the time alternative flavonolignans began to attract attention, silybin had already established a strong foothold in the literature, making it difficult for other compounds to gain comparable visibility or momentum. Notably, early *in vivo* studies, published in the same year as the first reports on silychristin and silydianin, consistently demonstrated hepatoprotective and antioxidant effects, thereby laying the groundwork for silybin’s continued investigation (Tuchweber et al., 1976).

From both technical and commercial standpoints, silybin has remained the most accessible component in silymarin extracts. Its relative ease of purification and availability in standardized pharmaceutical-grade preparations have made it a convenient and reliable choice for both experimental and clinical studies. In contrast, other flavonolignans occur in lower concentrations, and were historically more difficult to isolate in sufficient purity or quantity, posing a practical barrier to broader investigation. Importantly, the disproportionate research focus on silybin is not solely a reflection of its biological activity or availability, but also illustrates the systemic mechanisms by which scientific attention accumulates over time. Early recognition in a given research area can trigger a process of cumulative advantage, in which initial visibility leads to increased citations, funding, and infrastructure - further entrenching the prominence of that subject. This self-reinforcing feedback loop often favors already established topics, even when equally promising alternatives exist (David, 1994). In the context of silymarin research, this process likely contributed to a lock-in effect, whereby the scientific community became committed to silybin as the principal focus of investigation. The early availability of purified silybin, its incorporation into standardized formulations, and the rapid accumulation of supporting literature made it progressively more difficult to shift focus to other flavonolignans, even as compelling data on their potential began to emerge. This lock-in effect has also carried an opportunity cost, as research attention and funding have been disproportionately directed toward silybin, potentially delaying the identification of distinct pharmacological activities in other silymarin constituents. Over time, this dynamic likely constrained the broader scientific exploration of the silymarin complex.

Additionally, formulation development and clinical translation efforts have consistently favored silybin. The commercial success of products such as Legalon SIL and Realsil, both based on silybin derivatives, has further reinforced its status as the primary therapeutic agent within the silymarin complex. This has further incentivized scientists and pharmaceutical companies to continue investing in silybin-centric products, rather than exploring less known analogs.

**FIGURE 2:**
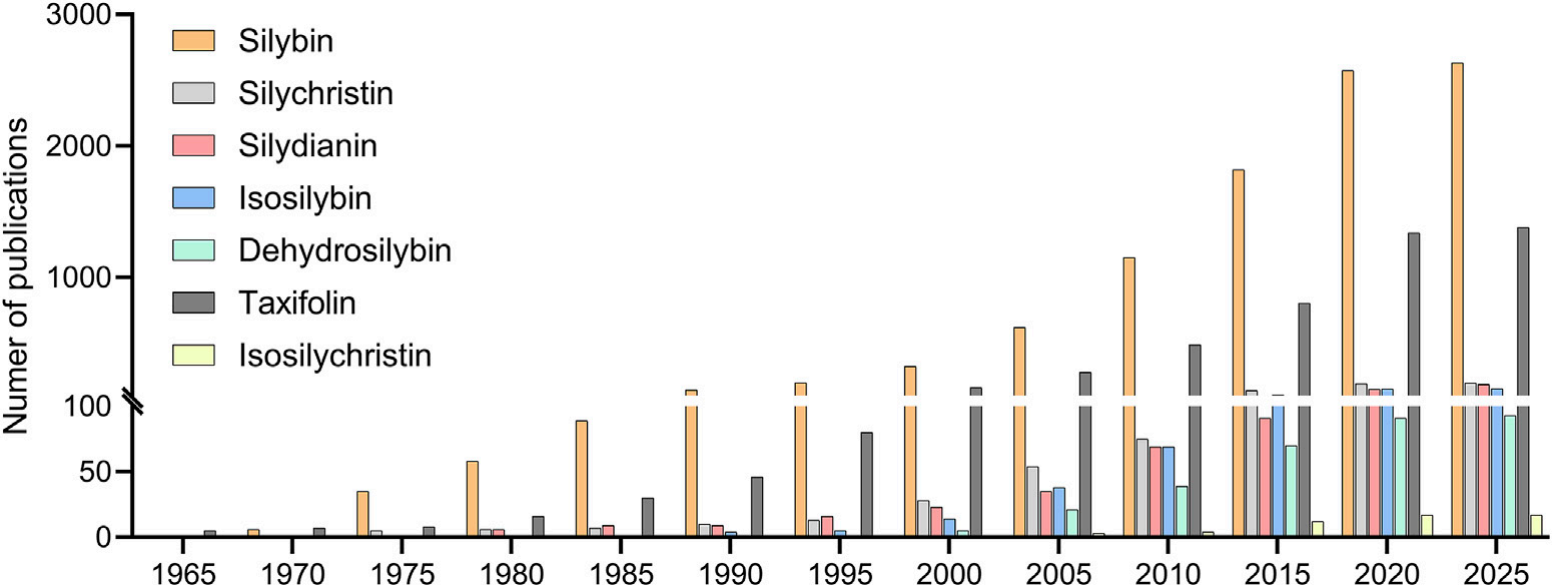
Cumulative number of PubMed-indexed publications (1960–2025) mentioning individual silymarin components. Publication counts reflect total keyword hits for: silybin, silychristin, silydianin, isosilybin (for both isomers), dehydrosilybin, taxifolin and isosilychristin. Data were obtained using single-term queries in PubMed on 31 March 2025.

In this context, the underrepresentation of other flavonolignans is not necessarily indicative of inferior biological activity, but rather reflects a combination of historical precedence, logistical convenience, and scientific conservatism, understood as the tendency of research communities to prioritize well-characterized and widely available compounds over less familiar ones. This trend is further evident in the scientific literature, where publication counts overwhelmingly favor silybin over other silymarin components. A keyword search on PubMed conducted in March 2025 revealed over 2,600 publications mention silybin, compared to 198 for silychristin, 188 for silydianin, 156 for isosilybins, 93 for dehydrosilybin, and only 17 for isosilychristin. Although taxifolin is present in many plant species beyond *Silybum marianum*, it has also been studied less extensively than silybin, with approximately 1,380 publications to date. This publication asymmetry underscores the dominance of silybin in silymarin research and the relative neglect of its structurally and functionally distinct counterparts (Figure 2).

## 4 The case for a broader perspective

Current advances in isolation, synthesis, and analytical methods now enable a systematic investigation of less known silymarin compounds. Recent studies have shown that individual compounds such as isosilybin A, silybin A (as an isolated diastereomer), taxifolin, and silychristin, exhibit promising pharmacological effects that, in some cases, surpass those of silybin (Deep et al., 2007; Polyak et al., 2010; Anthony and Saleh, 2013; Jurčacková et al., 2025). The therapeutic potential of these molecules remains largely underappreciated and underexplored. A more comprehensive evaluation of their pharmacological profiles may uncover previously overlooked benefits embedded within the silymarin complex - benefits that have long remained obscured by the prevailing dominance of silybin. Rather than continuing to treat silybin as the default and sole representative of silymarin’s bioactivity, future studies should aim to systematically evaluate the full spectrum of silymarin constituents, both individually and in combination, using rigorous and comparative approaches.

Beyond the inherent variability of crude silymarin, deliberate investigations on defined combinations of purified flavonolignans could reveal specific interaction profiles, whether synergistic, additive, or antagonistic, that remain largely unexplored. Such studies are not only valuable for understanding pharmacodynamic interactions but also for optimizing efficacy and tailoring therapeutic interventions to specific pathophysiological conditions. Further support for this broader perspective comes from recent *in vivo* evidence demonstrating the therapeutic potential of less known silymarin constituents. An ethyl acetate extract of *Silybum marianum* seeds, particularly rich in silydianin and silychristin (together accounting for over 50% of the extract), significantly improved multiple metabolic parameters in a rat model of metabolic syndrome—including body weight, glucose tolerance, triglyceride levels, and blood pressure—both in prevention and treatment protocols. Notably, the extract contained less than 8% silybin, suggesting that these effects were primarily mediated by non-silybin flavonolignans (Awla et al., 2023). This shift in perspective may also open the door to personalized medicine approaches, in which silymarin-derived formulations are adapted to individual patient needs, disease states, or even genetic variations affecting flavonoid metabolism. For instance, a taxifolin-enriched preparation could be advantageous in conditions marked by high oxidative stress, isosilybin-based combinations may be more effective against cancers and silybin A (as an isolated diastereomer) may be particularly beneficial in viral or inflammatory conditions.

Efficient drug delivery is a crucial but often overlooked factor in maximizing the therapeutic value of plant-derived compounds. Even small amounts of bioactive flavonolignans may achieve significant effects if properly delivered to target tissues. Nanoparticle-based delivery systems offer promising strategies for enhancing stability, bioavailability, and targeted cellular uptake. While most applications to date have centered on silybin, these platforms hold exceptional potential for underexplored constituents such as isosilybin B, silychristin, and silydianin. For instance, a nanoemulsion incorporating silydianin significantly enhanced anti-inflammatory activity *in vitro* (Tran et al., 2022), and silybin-coated gold nanoparticles were up to 4–5 times more cytotoxic against lung cancer cells than free silybin (Ravi et al., 2022). Such nanodelivery systems may help unlock the full therapeutic potential of silymarin constituents by improving their targeting precision and reducing the need for high systemic doses.

The increasing availability of purified standards, advanced isolation techniques, and cell- and mechanism-specific bioassays provides a solid foundation for this necessary shift. Moving beyond the current “silybin-centric” paradigm is not only scientifically justified, but essential for unlocking the full therapeutic potential of the silymarin complex. By adopting a broader perspective, we may not only deepen our understanding of silymarin as a phytocomplex, but also identify new candidates for drug development, nutraceutical innovation, or adjunctive therapies targeting hepatic or systemic diseases. A more nuanced exploration of these constituents will help bridge the gap between traditional herbal medicine and modern pharmacology, integrating the complexity of natural products into evidence-based therapeutic strategies.

## 5 Conclusions and future directions

Historically, silybin has come to dominate both experimental and clinical research on *Silybum marianum*. This predominance is understandable, given its abundance in silymarin, early structural understanding, and widespread clinical availability. In recent years, numerous studies have demonstrated that other constituents, such as isosilybin A, silybin A (as an isolated diastereomer), taxifolin, or silychristin, exhibit unique pharmacological properties compared to silybin. These include enhanced antioxidant activity, selective cytotoxicity against cancer cells, anti-inflammatory effects, and immunomodulatory potential. Despite these promising attributes, these molecules remain insufficiently explored, particularly for their pharmacokinetics, safety profiles, and clinical efficacy.

Looking ahead, greater emphasis on systematically exploring the pharmacological properties of individual flavonolignans beyond silybin should be placed on. Other constituents, such as isosilybins, silychristin, or dehydrosilybin, may exert superior biological effects in certain contexts. Comprehensive and comparative studies encompassing all major flavonolignans and taxifolin are warranted. Despite the challenges, such as requiring extraordinary time and resources, these investigations could clarify structure–activity relationships and identify the most promising therapeutic candidates among silymarin constituents.

Particularly valuable would be studies comparing these compounds side by side in disease models, including cancer, viral infections, and metabolic disorders, where some of these molecules have already shown promising activity. Additionally, future research should investigate pharmacokinetic and metabolic properties to determine differences in bioavailability and tissue distribution, especially as these factors strongly influence *in vivo* efficacy. A recent study has shown that individual silymarin constituents differ markedly in their pharmacokinetic profiles even when administered as part of the same extract. Silybin, isosilybin A + B, and silychristin exhibit distinct absorption kinetics, plasma exposure levels, and elimination rates in mice (Ralli et al., 2023). Moreover, modern pharmacokinetic methodologies, such as sensitive ultra-high-performance liquid chromatography, have enabled detailed profiling of plant-derived bioactive compounds (Shang et al., 2024). These methodological advances support the workability of conducting rigorous pharmacokinetic studies on individual silymarin constituents in preclinical models. Ultimately, early-phase clinical trials targeting non-silybin flavonolignans, either individually or in optimized combinations, will be critical for translating preclinical findings into clinical benefit.

While silybin’s pharmacological importance is indisputable, it is time to look beyond this single molecule and rediscover the untapped therapeutic potential embedded within the broader silymarin complex. A shift in research attention could ultimately lead to the development of more effective and targeted therapies derived from the full spectrum of silymarin components.

## Data Availability

The original contributions presented in the study are included in the article/supplementary material, further inquiries can be directed to the corresponding author.
